# Discovery of High‐Performing Metal–Organic Frameworks for On‐Board Methane Storage and Delivery via LNG–ANG Coupling: High‐Throughput Screening, Machine Learning, and Experimental Validation

**DOI:** 10.1002/advs.202201559

**Published:** 2022-05-07

**Authors:** Seo‐Yul Kim, Seungyun Han, Seulchan Lee, Jo Hong Kang, Sunghyun Yoon, Wanje Park, Min Woo Shin, Jinyoung Kim, Yongchul G. Chung, Youn‐Sang Bae

**Affiliations:** ^1^ Department of Chemical and Biomolecular Engineering Yonsei University 50 Yonsei‐ro, Seodaemun‐gu Seoul 03722 South Korea; ^2^ School of Chemical and Biomolecular Engineering Georgia Institute of Technology Atlanta GA 30332 USA; ^3^ School of Chemical Engineering Pusan National University Busan 46241 South Korea; ^4^ Korea Institute of Industrial Technology 55 Joga‐ro, Jung‐gu Ulsan 44413 South Korea

**Keywords:** adsorbed natural gas, grand canonical Monte Carlo, high‐throughput screening, LNG–ANG coupling, metal–organic frameworks, methane storage, molecular dynamics simulation

## Abstract

Liquefied natural gas (LNG) gasification coupled with adsorbed natural gas (ANG) charging (LNG–ANG coupling) is an emerging strategy for efficient delivery of natural gas. However, the potential of LNG–ANG to attain the advanced research projects agency‐energy (ARPA‐E) target for onboard methane storage has not been fully investigated. In this work, large‐scale computational screening is performed for 5446 metal–organic frameworks (MOFs), and over 193 MOFs whose methane working capacities exceed the target (315 cm^3^(STP) cm^−3^) are identified. Furthermore, structure–performance relationships are realized under the LNG–ANG condition using a machine learning method. Additional molecular dynamics simulations are conducted to investigate the effects of the structural changes during temperature and pressure swings, further narrowing down the materials, and two synthetic targets are identified. The synthesized DUT‐23(Cu) and DUT‐23(Co) show higher working capacities (≈373 cm^3^(STP) cm^−3^) than that of any other porous material under ANG or LNG–ANG conditions, and excellent stability during cyclic LNG–ANG operation.

## Graphical abstract

Starting from 5446 structures without open metal sites (OMSs) in computation‐ready experimental (CoRE) metal–organic framework (MOF) database, grand canonical Monte Carlo (GCMC) simulations are conducted for 2144 structures to establish the top 50 materials with the highest working capacities in liquefied natural gas‐adsorbed natural gas (LNG–ANG) condition. By excluding the structures showing large structural changes during temperature swing from molecular dynamics (MD) simulations, DUT‐23(M) with superior LNG–ANG working capacity is experimentally verified.

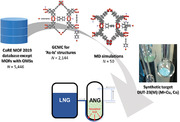

## 1 Introduction

Natural gas (NG) mostly comprises methane and is widely considered as a mid‐ to long‐term alternative transportation fuel because of its abundant reserves and relatively low environmental footprint compared with gasoline. However, the utility of NG as an onboard transportation fuel is limited because the storage of NG requires multistage compression up to a very high pressure (≈250 bar for compressed NG (CNG)) or a cryogenic temperature bath to maintain a very low tank temperature (≈120 K for liquefied NG (LNG)).^[^ ^1^ ^]^

To store and transport NG under relatively moderate conditions for practical applications, adsorbed NG (ANG) has been suggested as a viable alternative to overcome the limitations of both CNG and LNG.^[^ ^2^ ^]^ The approach using ANG involves filling the gas tank with porous adsorbent materials that can readily adsorb methane molecules at relatively low pressure (≈65 bar) and moderate temperature (≈298 K) compared with those using CNG and LNG, respectively. Thus, the cost of compressing the gas at high pressure is reduced and maintaining the tank temperature near the cryogenic temperature is unnecessary. Because of these advantages,^[^ ^3^ ^]^ ANG has shown competitive methane storage capacity compared with CNG (assuming ideal packing of adsorbents).^[^ ^1^, ^4^ ^]^ Accordingly, the design and discovery of new porous materials with high methane uptakes are important research fields to realize adsorptive NG storage systems.^[^ ^3b^ ^]^

Metal–organic frameworks (MOFs) are a class of crystalline porous materials that generally constitute repeated coordination of inorganic clusters and organic ligands.^[^ ^1^, ^3^, ^5^ ^]^ With appropriate selection of ligand and metal precursors, it is possible to synthesize a large number of porous MOF structures with various physical and chemical properties.^[^ ^6^ ^]^ MOFs have been employed for various applications including gas storage and separation, catalysis, and energy applications.^[^ ^7^ ^]^ However, the number of porous MOFs synthesized to date exceeds 14 000^[^ ^8^ ^]^; thus, it is difficult to assess the properties of these materials via synthetic approaches alone.

Computational high‐throughput screening is a powerful approach that can be used to rapidly evaluate the performance of a large number of materials for a given application.^[^ ^5^, ^9^ ^]^ Considering MOFs for NG storage,^[^ ^23^ ^]^ high‐throughput screening of 650 000 structures including experimental and hypothetical structures of MOFs, zeolites, and porous polymer networks for ANG application indicated the difficulty in achieving the target metric of the working capacity (315 cm^3^(STP) cm^−3^) proposed by the Advanced Research Projects Agency‐Energy (ARPA‐E) of the US Department of Energy (DOE), rendering a controversial verdict on porous material‐based methane storage and delivery.^[^ ^10^ ^]^

**Figure 1:**
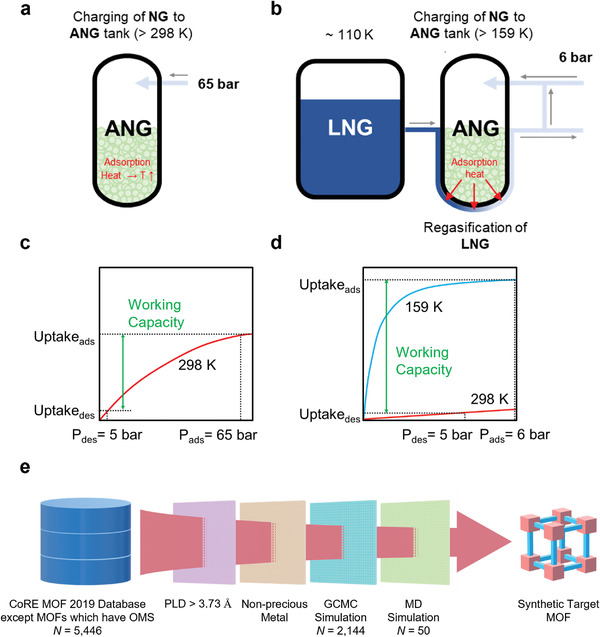
Schematics of a) typical adsorbed natural gas (ANG) charging process and c) isotherm, b) ANG charging process coupled with liquefied natural gas (LNG) regasification process (i.e., LNG–ANG coupling) and d) isotherms, e) hierarchical computational screening procedure for reducing the number of structure candidates (*N*) for the LNG–ANG system from CoRE metal–organic framework (MOF) database through combined grand canonical Monte Carlo (GCMC) and molecular dynamics (MD) simulations.

Recently, the concept of LNG–ANG coupling, which combines LNG gasification and ANG charging processes, has been suggested in the literature as a promising method for storing methane.^[^ ^11^ ^]^ In LNG–ANG coupling, a large working capacity can be achieved by decreasing the charging temperature of the ANG process to ≈160 K as a result of heat exchange with low‐temperature LNG. In this scenario, the tank is filled at a substantially lower pressure than that in the ANG scenario and does not require expensive multistage compressors for charging (**Figure** **1**a,b). The approach has two main advantages: first, more methane can be stored at a significantly lower pressure (Figure 1c,d); and second, the LNG–ANG coupling system does not require additional equipment that is often necessary for cooling the ANG tank, because the generated heat of adsorption can be delivered to the LNG gasification process and quickly exhausted for vaporizing LNG.^[^ ^11c^ ^]^ Despite these advantages, the potential of LNG–ANG coupling to achieve the ARPA‐E target for onboard methane storage and delivery has not been studied.^[^ ^12^ ^]^

In this work, we used high‐throughput computational screening to identify high‐performance MOFs from the 5446 structures without open metal site (OMS) in the Computation‐ready Experimental (CoRE) MOF database.^[^ ^8^ ^]^ After simple two‐step filter based on the pore size and the identity of metal species, high‐throughput grand canonical Monte Carlo (GCMC) simulations were conducted for 2144 structures to establish the top 50 materials with the highest working capacities in the LNG–ANG condition. LNG–ANG operations require a large temperature swing of ≈140 K between desorption and adsorption operation that could lead to the changes in the lattice parameters of the crystal structures and the available pore volumes. The degree of changes could be significant especially for the highly porous and flexible materials such as MOFs. Molecular dynamics (MD) simulations were carried out on 50 structures to identify materials that show minimal changes in the overall structure with respect to the large temperature change. Subsequent GCMC simulations on the structure obtained from the MD simulation snapshots were able to identify materials with minor changes in their methane uptakes and working capacities during the temperature and pressure swings. Machine learning (ML) model was trained on the large data from the simulation to compare the changes in the feature importance of the material's performance between the LNG–ANG and ANG conditions. Finally, simulation predictions were validated by synthesizing and testing DUT‐23(M) (M = Cu, Co) for methane storage and delivery capacities under the LNG–ANG condition, and the performances of these materials were found to exceed the performance target set by the ARPA‐E for onboard methane storage and delivery.

## 2 Results

### 2.1 Screening of the CoRE MOF Database for LNG–ANG and ANG Conditions

Large‐scale GCMC simulations were carried out under two conditions: one for the LNG–ANG coupling system and for an ANG system. For the LNG–ANG system, the adsorption (charging) of methane was assumed to be carried out at 6 bar and 159 K, while desorption (discharging) was carried out at 298 K and 5 bar.^[^ ^24^ ^]^ For the ANG system, GCMC simulations were carried out at 65 and 5 bar for adsorption and desorption conditions at 298 K, respectively.^[^ ^3a^ ^]^ The methane working capacity is the difference between the methane uptake at the adsorption condition and the methane uptake at the desorption condition.^[^ ^13^ ^]^

**Figure 2:**
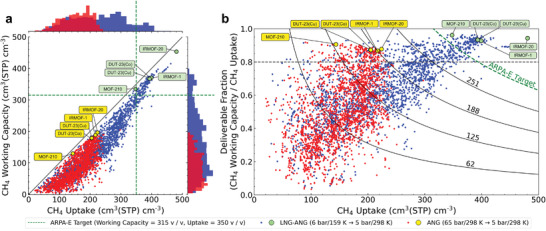
Results of high‐throughput screening for the structures without OMS from the CoRE MOF 2019‐ASR Database (*N* = 2144) for LNG–ANG and ANG systems. Each point corresponds to data from a single MOF structure. Blue points and green circles represent simulation data from the LNG–ANG condition (159 K, 6 bar adsorption → 298 K, 5 bar desorption). Red points and yellow circles indicate simulation data from ANG condition (298 K, 65 bar adsorption → 298 K, 5 bar desorption). Green dashed lines highlight the ARPA‐E targets (working capacity target: 315 cm^3^(STP) cm^−3^, uptake target: 350 cm^3^(STP) cm^−3^).

**Figure** **2** shows the results of the high throughput computational screening. In general, higher methane working capacities are accomplished under the LNG–ANG condition compared to that of the ANG condition. Under the ANG condition, nearly all of the structures exhibited methane working capacities less than 200 cm^3^(STP) cm^−3^, which were consistent with the results from Simon and coworkers.^[^ ^22^ ^]^ Simon and coworkers considered increasing the adsorption pressure to increase the methane uptake in the system while increasing the desorption temperature to desorb all stored methane to improve the performance of ANG, but were not able to achieve a methane working capacity above 250 cm^3^(STP) cm^−3^. In contrast, the LNG–ANG coupling approach adopted here takes an inverse approach where the methane uptake in the system was increased by lowering the charging temperature. Under the LNG–ANG condition, we identified 732 structures with methane working capacities higher than 200 cm^3^(STP) cm^−3^. Among the top‐performing materials from the computational screening under the LNG–ANG condition, IRMOF‐20 (CSD REFCODE: VEBHUG) has the highest working capacity of 453 cm^3^(STP) cm^−3^, which is twice as high as the highest value of the methane working capacity under ANG conditions.

Further analysis of the molecular simulation data demonstrated the practical advantage of using the LNG–ANG coupling system. Under the conventional ANG condition, we found that nearly 62.6% of the CoRE MOF structures without OMS have higher working capacities than that of an empty tank (i.e., 62 cm^3^(STP) cm^−3^). Meanwhile, under LNG–ANG coupling, 83.5% of the CoRE MOF structures without OMS exhibited higher working capacities than the 62 cm^3^(STP) cm^−3^. This indicates that the low charging temperature of the LNG–ANG coupling system which favors physisorption^[^ ^6a^ ^]^ leads to more efficient storage and delivery of methane compared to the ANG system. We also found that 25.0% of the structures could deliver more than 80% of the stored methane under the LNG–ANG condition, whereas only 11.4% of the structures were able to do this under ANG conditions (Figure 2b). Remarkably, under the LNG–ANG condition, 9.0% (193 structures) of the MOFs surpassed the volumetric working capacity target set by the ARPA‐E of the US DOE (315 cm^3^(STP) cm^−3^), which is very challenging to achieve based on ANG alone. In addition, all these structures can deliver more than 80% of the stored methane. Thus, LNG–ANG coupling is more promising than the typical ANG approach for an onboard methane storage and delivery system. In terms of the gravimetric working capacity target, 2.71% (58 structures) of MOFs surpassed the ARPA‐E target (0.5 g g^−1^) (Figure S1, Supporting Information).

**Figure 3:**
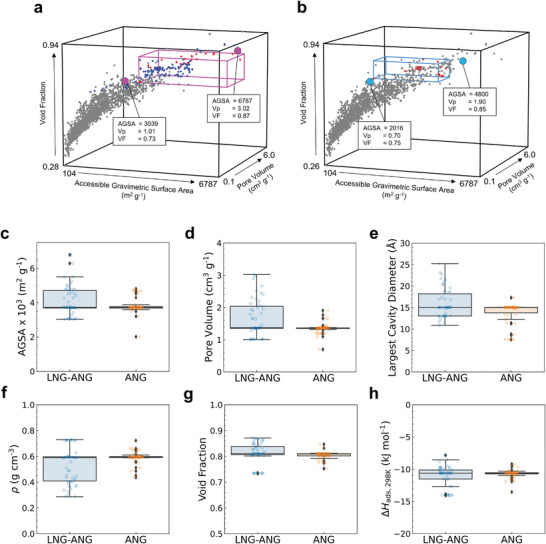
Structure–performance relationships for the methane working capacity, accessible gravimetric surface area (AGSA), void fraction (VF), and pore volume (*V*_p_) under a) LNG–ANG condition (6 bar, 159 K → 5 bar, 298 K) and b) ANG condition (65 bar, 298 K → 5 bar, 298 K). Boxes highlight the properties of the top 50 materials regarding methane working capacity. Red points indicate the properties of the top 50 materials under both conditions. Blue points in (a) show the properties of the structures achieving the ARPA‐E target. Boxplots of c) AGSA, d) *V*_p_, e) largest cavity diameter (LCD), f) density, g) VF, and h) heat of adsorption for structures indicated with the orange points under ANG and with the blue points under LNG–ANG conditions. The horizontal line in each rectangular box represents the median value (second quartile) of the group, while the lower and upper boundaries of the box correspond to the first and third quartiles, respectively. The position of the upper/lower bar is set to the smallest/largest of the maximum/minimum value or upper/lower boundary +/− 1.5 × IQR (IQR = value of third quartile − value of first quartile), respectively. If the maximum/minimum value is outside of the bars, it is called an outlier. Such values are represented by the dots.

**Figure** **3** displays the structure‐property relationship comparisons based on the simulated data to delineate the differences in the structural features for optimal materials under the two conditions. In general, the top 50 materials in terms of methane working capacity for the LNG–ANG condition tend to be possess higher porosity (AGSA and *V*_p_) than those under the ANG condition (Figure 3a,b). Notably, the range of the pore volume in the top 50 structures significantly shifted from 0.70–1.90 cm^3^ g^−1^ in ANG to 1.01–3.02 cm^3^ g^−1^ in LNG–ANG. The data show that the design and synthesis of highly porous materials is optimal under the LNG–ANG conditions. Additional analyses show that the LNG–ANG condition requires larger pores and lighter materials than the ANG condition (Figure 3c–g). Figure 3e show the LCDs for materials with top working capacities under the LNG–ANG condition were larger than those under the ANG condition. Furthermore, the materials with the highest working capacities under the LNG–ANG condition had relatively lower crystal densities (Figure 3f). The boxplots highlight specific ranges of properties favored for the top‐rated LNG–ANG adsorbents. Additional structural features that optimize the performance under LNG–ANG condition includes adsorbents with large micropores (13.1 Å < LCD < 18.2 Å) and high surface areas (3688 m^2^ g^−1^ < AGSA < 4711 m^2^ g^−1^) (Table S3, Supporting Information).

Machine learning (ML) model was developed to quantify the relative importance of the physical and adsorptive properties on the performance of MOFs under different operating conditions (Figure S2, Supporting Information). Gradient boosting regressor (GBR) model was trained on the data from high‐throughput screening. The accessible gravimetric surface area (AGSA) was identified as the most important feature for determining methane uptake than any other structural property at charging for both ANG and LNG–ANG. In contrast, at the discharging conditions of 298 K and 5 bar, the relative importance of the AGSA was lower (15%) than that of the heat of adsorption (Δ*H*_ads_) (36%). Moreover, the relative importance of the void fraction (VF) was large for methane uptake under both charging and discharging conditions. On the basis of these results, AGSA is the most important feature for obtaining high methane working capacity under ANG conditions followed by the VF and Δ*H*_ads_ which is in line with previous high‐throughput computational screening results.^[^ ^10^, ^14^ ^]^ Top most important features for the LNG–ANG condition are the same as those for the ANG condition, except the importance of the VF is larger for the LNG–ANG system. Developed ML model and the feature importance analyses show that the materials that showed good performance under ANG condition will likely be high‐performing materials under the LNG–ANG condition.

### 2.2 Effect of Operating Conditions on Structure and Performance

MOFs are relatively flexible materials compared with other classes of crystalline porous materials (such as zeolites), so their underlying structural properties may be altered from to the large temperature swing (≈140 K) during charging/discharging operation for the LNG–ANG method. The structures in the CoRE MOF database were constructed based on the geometries experimentally measured via single‐crystal X‐ray diffraction (XRD) at various temperatures and pressures. Some of the structures were geometrically minimized after the solvent removal steps, but the lattice parameters (unit cell lengths and angles) were kept the same at the experimentally reported values. Therefore, the lattice parameters of these structures are subject to change at the temperature and pressure at which adsorption occurs. However, computational screening studies thus far only used the geometry of structures presented in the CoRE MOF database without the consideration of the structural changes due to the external temperature or pressure. Framework flexibility is one of the three reasons that the simulation and experimental isotherms do not match.^[^ ^15^ ^]^ For a more accurate simulation, the structures could be relaxed using MD simulation to reach equilibrium geometries at the target temperature or pressure.

To validate the performances of materials considering the possible structural changes under the LNG–ANG condition, MD simulations were performed for the top 50 materials selected from the screening of the original “As‐Is” CoRE MOF structures. The calculated pore size distributions (PSDs) of the structures after the simulations revealed that the pores generally shrunk at 6 bar and 159 K, in terms of both the largest pore diameter and smallest pore diameter (Figures S3a,b, Supporting Information). From the PSD plots, the most frequent pore size of the original structures were 14.4 Å, but that of the MD‐relaxed structures was much smaller (10.5 Å) (Figure S3d, Supporting Information).

**Figure 4:**
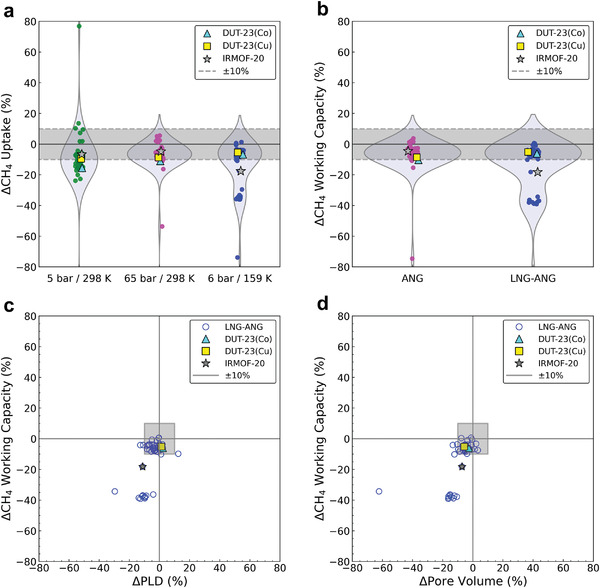
Percentage changes in a) methane uptakes and b) methane working capacities of the top 50 materials from high‐throughput screening after MD simulations at different operating conditions. Violin plots show distributions of percentage differences for the methane uptakes and working capacities. All violin plots have equal widths. The *x*‐axes of (c) and (d) are the percentage changes in the structural properties of the top 50 materials from high‐throughput screening after the MD simulation at 6 bar and 159 K. The *y*‐axes of (c) and (d) are the percentage changes in the methane working capacities under the LNG–ANG condition.

Using the relaxed structures obtained from the MD simulations at the temperatures and pressures of interest, GCMC simulations were performed to obtain methane uptakes and working capacities under LNG–ANG and ANG conditions. Methane uptake generally decreases at high pressures (65 bar, 298 K) and low temperatures (6 bar, 159 K) (**Figure** **4**). However, at 5 bar and 298 K, the decrease in the methane uptake is not significant, and many materials exhibit increased methane uptake (Figure 4a). As a result, the methane working capacities generally decreased under both ANG and LNG–ANG conditions (Figure 4b). The number of structures with large decreases in working capacities (>20%) was higher in the LNG–ANG condition (12 out of 50) than in the ANG condition (2 out of 50), which directly shows that the consideration of structural changes in MOFs is essential to model systems under large temperature changes. In most cases, the materials with large decreases in the methane working capacities under the LNG–ANG condition have smaller pore sizes, smaller surface areas, smaller void fractions, larger heats of adsorption, or larger densities compared with their original structures (Figure 4c,d; Figure S4, Supporting Information), which is similar to the characteristic of the LNG–ANG condition derived from the structure–performance analysis from ML model. In this context, the reason that the working capacities in the LNG–ANG condition generally decrease after MD is likely due to the decrease in the largest pore diameters below the optimal pore diameter (<13.1 Å) under the LNG–ANG condition (Table S3 and Figure S3, Supporting Information).

MOFs for synthetic targets were identified based on the methane adsorption characteristics considering the structural changes due to the external temperature and pressure. Because the performances of the original structures of the top 50 materials were already far above the ARPA‐E target, we focused on materials that show minimal changes in their methane uptakes and working capacities. This is also because the MOFs with high flexibility might be vulnerable to the reduction in crystallite size, partial collapse of the pore structure, and deformation of their adsorptive characteristics. MD simulations could help to remove such potentially problematic structures. For example, the structural properties and working capacity of IRMOF‐20 (VEBHUG), the best material in terms of the methane working capacity under LNG–ANG condition, significantly changed after MD (working capacity decreased by 18.2%); therefore, IRMOF‐20 was not selected as a synthetic target (Figure 4). Under the LNG–ANG condition, 24 out of 50 MOFs displayed small changes (within ±7%) in their methane uptakes, while 27 out of 50 MOFs showed small changes (within ±7%) in their methane working capacities, with 24 overlapping MOF structures. Among the 24 MOFs, we identified DUT‐23(Co) and DUT‐23(Cu) as viable synthetic targets considering stability and commercial availability of ligands. In addition, most of the structural properties (i.e., pore‐limiting diameter (PLD), AGSA, VF, Δ*H*_ads_, and pore volume) of DUT‐23(Co) and DUT‐23(Cu) changed within ±10% after the MD simulations, making these materials suitable candidates for LNG–ANG adsorbents with high performance and rigid structures (Figure 4c,d; Figure S4, Supporting Information). Only the LCDs of DUT‐23(Co) and DUT‐23(Cu) changed considerably, but after MD relaxation, their LCDs were within the optimal region (13.1 Å < LCD < 18.2 Å) for LNG–ANG (Table S3 and Figure S5, Supporting Information).

### 2.3 Synthesis and Evaluation of DUT‐23(M)

**Figure 5:**
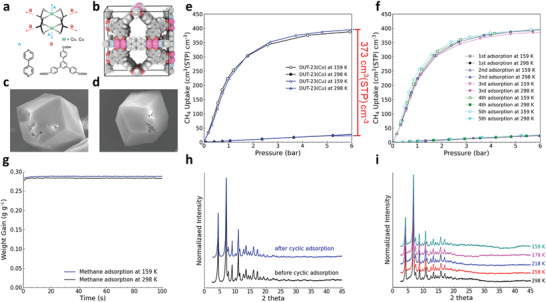
a) Inorganic and organic building units of DUT‐23(Co) and DUT‐23(Cu). b) Pore structure of DUT‐23(Co) and DUT‐23(Cu) in the CoRE MOF database. SEM images of DUT‐23(Cu) crystals c) before and d) after 5 cycles of temperature swing adsorption. e) Methane uptakes of synthesized DUT‐23(Co) and DUT‐23(Cu) at 159 and 298 K up to 6 bar, and f) methane isotherms of DUT‐23(Cu) obtained during 5 cycles of stability test. At the beginning of each cycle, methane was adsorbed into a degassed sample at 159 K up to 6 bar. After a short degassing at 298 K of approximately 10 min, methane adsorption at 298 K up to 6 bar was conducted. g) Normalized weight gains of DUT‐23(Cu) during a methane dosing step to establish approximately 0.28 g g^−1^ of methane. h) XRD patterns of DUT‐23(Cu) measured before and after the cyclic adsorption experiment. i) In situ XRD of DUT‐23(Cu) measured at 298, 258, 218, 178, and 159 K.

DUT‐23(Co) (ICAQIO) and DUT‐23(Cu) (ICAQOU) were synthesized based on reported synthesis procedures,^[^ ^16^ ^]^ from divalent metal salts and two types of ligands (**Figure** **5**a,b), to form MOFs with pto topologies and two distinct pores (8 and 20 Å). The powder XRD (PXRD) patterns of the synthesized samples match the PXRD patterns calculated from the crystal structures (Figure S6, Supporting Information), suggesting that the synthesized samples are close to the defect‐free crystal structures used in the simulation. Nitrogen adsorption of the synthesized samples at 77 K revealed that the samples have similar adsorption behaviors to those calculated from the GCMC simulations (Figure S7, Supporting Information). Moreover, the BET surface areas of the samples calculated from the experimental N_2_ isotherms were 5185 and 5175 m^2^ g^−1^ for DUT‐23(Co) and DUT‐23(Cu), respectively, which are almost the same as the BET surface areas calculated from the simulated N_2_ isotherms (5168 and 5152 m^2^ g^−1^, respectively) (Figures S8, S9 and Table S4, Supporting Information). Finally, the extraordinary capabilities of DUT‐23(Co) and DUT‐23(Cu) to deliver methane predicted via the simulations were validated by the experimental methane adsorption isotherms of the synthesized samples (Figure 5e). Under both equilibrium conditions in LNG–ANG, 6 bar at 159 K and 5 bar at 298 K, the experimental methane uptakes of the samples were slightly lower than the values from the GCMC simulations (Figure S10, Supporting Information). The small deviations might be due to imperfect crystallinity of the samples and/or slight shrinkage in the pores of the samples under low‐temperature conditions. The differences between the experimental and simulation results were even smaller in terms of the working capacities (**Table** **1**). Specifically, the experimental working capacities were 365.3 and 373.1 cm^3^(STP) cm^−3^ for DUT‐23(Co) and DUT‐23(Cu), respectively. To the best of our knowledge, these working capacities under the LNG–ANG condition are higher than those of any other porous material in either ANG or LNG–ANG conditions and are significantly higher than the ARPA‐E target (315 cm^3^(STP) cm^−3^). At both 298 and 159 K, the adsorption equilibrium of methane on DUT‐23(Cu) was quickly achieved within a few seconds (Figure 5g), implying that the large pore size of DUT‐23(Cu) provides fast mass transport of methane even at low temperatures.

**Table 1:** Experimental methane uptakes, working capacities, and BET surface areas of selected MOFs for the LNG–ANG and ANG systems

Structure	Methane uptake [cm^3^(STP) cm^−3^]			Working capacity [cm^3^(STP) cm^−3^]		BET surface area [m^2^ g^−1^]	Ref.
	6 bar, 159 K	65 bar, 298 K	5 bar, 298 K	LNG–ANG	ANG		
DUT‐23(Co)	387.3 (397.2)	200.1 (205.0)	22.0 (26.5)	365.3 (370.6)	178.1 (178.5)	5185 (5168)	This work
DUT‐23(Cu)	395.1 (393.9)	205.4 (203.3)	22.0 (26.8)	373.1 (367.1)	183.4 (176.5)	5175 (5152)	This work
MIL‐53(Al)	311.8	186.8	49.5	262.3	137.3	1223	[11b]
MIL‐101(Cr)	275	215	35.0	240	180	3302	[11a]
HKUST‐1	324.1*	263.8	75.0	249.1*	188.8	1850	[3b]

The data in the parentheses are simulated values;

*The data for HKUST‐1 was calculated with a charging condition of 6 bar and 200 K.

One possible issue of the LNG–ANG method is that the large change in the temperature during an adsorption–desorption cycle may impact the performance of the adsorbent materials because of the possible shrinkage and swelling of the framework.^[^ ^17^ ^]^ To test the stability under the LNG–ANG condition, we performed five cycles of adsorption on DUT‐23(Cu) with changing temperature between 159 and 298 K (Figure 5f). For each cycle, the methane adsorption isotherm of DUT‐23(Cu) was obtained up to 6 bar at 159 K, and after 1 h of evacuation, the methane adsorption isotherm of the sample was measured again up to 6 bar at 298 K. Remarkably, the consecutive adsorption/desorption at cryogenic and room temperatures did not decrease the methane uptake of the sample. In addition, the crystallinity of DUT‐23(Cu) was maintained after consecutive adsorption/desorption (Figure 5c,d,h). This could be due to the rigid characteristic of DUT‐23(Cu) during the temperature and pressure swings, which was confirmed through the additional MD simulation steps during the screening. The rigid characteristic of DUT‐23(Cu) during the large temperature change was also verified by the in situ XRD patterns, recorded at temperatures between 298 and 159 K (Figure 5i).

## 3 Discussion

In this work, high‐throughput computational screening was carried out to screen the CoRE MOF 2019 database for methane storage and delivery application under the LNG–ANG condition. The high‐throughput screening exercises were able to identify 193 structures with higher working capacities than the ARPA‐E target under the LNG–ANG condition. An in‐depth analysis and machine learning model based on the data from computational screening suggested that the AGSA, VF, and heat of adsorption are key features for optimizing methane storage and delivery under LNG–ANG conditions. The more accurate methane uptake of top‐performing materials under the operating conditions of LNG–ANG was investigated based on MD simulations to narrow down promising materials for target synthesis, and two isostructural MOFs (DUT‐23(M), M = Cu, Co) were identified as promising materials and synthesized. According to the structural analyses and gas adsorption experiments, the structural and adsorptive characteristics of the synthesized adsorbents agreed well with the simulation predictions. As a result, the methane working capacities, predicted to be much higher than the ARPA‐E target via simulation, were experimentally validated. Thus far, to the best of our knowledge, the measured methane working capacities of DUT‐23(Cu) and DUT‐23(Co) (373.1 and 365.3 cm^3^(STP) cm^−3^, respectively) under the LNG–ANG condition were higher than that of any other porous material reported under either ANG or LNG–ANG condition. Furthermore, DUT‐23(Cu) was stable under repeated methane adsorption under the LNG–ANG condition, which was also partially predicted from its rigid characteristic in the MD results. Employing a novel computational screening strategy considering the effects of external temperature and pressure on the MOF structures, we obtained record‐high‐performance materials for the LNG–ANG coupling system, which is expected to be used in practice for onboard methane storage and delivery.

## 4 Experimental Section

### Dataset Screening Steps

In this work, the internal version all‐solvent removed (ASR) version of the CoRE MOF 2019 database was used.^[^ ^8c,d^ ^]^ Several filters including the pore size and the identity of the metal atoms were used to initially reduce the 14142 MOFs (Figure 1e). First, 8696 structures were excluded with OMS because it was known that general force fields did not accurately reproduce the interaction between OMS and methane molecule. Second, the PLDs for all structures using Zeo++ package were calculated^[^ ^25^ ^]^ and excluded structures with PLDs smaller than 3.73 Å, which was the van der Waals (vdW) diameter of a methane model from TraPPE force field.^[^ ^18^ ^]^ If the PLD was smaller than 3.73 Å, access of the methane molecule to the pore was considered to be difficult because of the narrow window size. Structures containing precious metal atoms (gold, silver, dysprosium, europium, gallium, gadolinium, hafnium, indium, iridium, lanthanum, molybdenum, neodymium, palladium, praseodymium, platinum, rhodium, ruthenium, selenium, samarium, terbium, tellurium, thulium, uranium, and yttrium) were excluded to determine industrially applicable targets. Grand canonical Monte Carlo (GCMC) simulations were carried out to predict the methane uptake on the remaining 2144 structures at the charging conditions of the LNG–ANG and ANG processes (6 bar, 159 K, and 65 bar, 298 K, respectively) and discharging conditions (5 bar, 298 K). Top 50 materials in terms of working capacity under LNG–ANG conditions were subjected to isothermal‐isobaric ensemble (NPT) MD simulations for 1 ns for materials. Final configurations from the NPT MD simulations were then energy minimized under the Canonical (NVT) ensemble while keeping the lattice constant, and GCMC simulations were subsequently carried out for the energy‐minimized configurations.

### Molecular Modeling

GCMC simulations were performed to discover the top‐performing MOFs for the LNG–ANG condition. The Lennard–Jones (LJ) 12–6 potential was used to approximate the van der Waals interaction between adsorbate–adsorbate and adsorbate–framework atomic interactions (Equation (1)):

(1) $$U_{\mathrm{ij}}=4\varepsilon_{\mathrm{ij}}\left[{\left(\frac{\sigma_{\mathrm{ij}}}{r_{\mathrm{ij}}}\right)}^{12}-{\left(\frac{\sigma_{\mathrm{ij}}}{r_{\mathrm{ij}}}\right)}^{6}\right]$$

where *U_ij_* is the interaction energy between atoms *i* and *j*, and *r_ij_* is the distance between the two atoms; *ε*_*ij*_ and *σ*_*ij*_ are the LJ parameters that correspond to the LJ well depth and atomic size, respectively. Considering the purpose of the screening that rapidly derived high‐performing materials from a vast number of materials, the use of the general force field was essential.^[^ ^19^ ^]^ In a previous study, the uncharged TraPPE‐UA force field for methane in combination with the mixed DREIDING/UFF force field for the MOF atoms showed the best agreement with experiments among the common potentials, even for the methane adsorption on the MOFs with open metal sites.^[^ ^20^ ^]^ Therefore, in this study, the LJ parameters of framework atoms were obtained from the DREIDING force field. If the atom types were not available in the DREIDING force field, the universal force field (UFF) was used (Table S5, Supporting Information).^[^ ^21^ ^]^ Those of the methane molecule were obtained from the TraPPE‐UA force field, which regarded methane molecules as one interaction site (Table S6, Supporting Information).^[^ ^18^ ^]^ Nitrogen molecule was modeled as a rigid three‐sites with charges, and its LJ parameters were obtained from the TraPPE force field.^[^ ^22^ ^]^ The combination of TraPPE and DREIDING‐UFF had been used extensively in the literature, as described in several computational screening studies.^[^ ^9^, ^10^, ^23^ ^]^ To determine the LJ parameters for the interaction between different types of atoms, the Lorentz–Berthelot combining rule was used (Equations (2) and (3)):

(2) $$\varepsilon_{\mathrm{ij}}=\sqrt{\varepsilon_{\mathrm{ii}}\varepsilon_{\mathrm{jj}}}$$

(3) $$\sigma_{\mathrm{ij}}=\left(\sigma_{\mathrm{ii}}+\sigma_{\mathrm{jj}}\right)/2$$

The van der Waals interactions between atoms beyond the cutoff distance of 12.8 Å were not considered. To screen for methane uptake, GCMC simulations were conducted for 2500 initialization cycles followed by 2500 production cycles, in which insertion, deletion, translation, and reinsertion moves were used with equal probabilities. A cycle corresponded to either 20 or the number of adsorbate molecules in the system. Additional GCMC simulations were performed for 10 000 initialization cycles followed by 10 000 production cycles for the structures of the top 50 working capacities in high‐throughput computational screening. During the simulations, all framework atoms did not move from their reported crystallographic positions. For N_2_ adsorption isotherms at 77 K in DUT‐23(Cu) and DUT‐23(Co), GCMC simulations were carried out for 10 000 initialization cycles followed by 50 000 production cycles.

The heat of adsorption of each structure was calculated using 5000 cycles of the Widom insertion method at infinite dilution conditions at 298 K. The heat of adsorption is defined as

(4) $$\Delta H=\left\langle U_{\mathrm{hg}}\right\rangle -\left\langle U_{h}\right\rangle -\left\langle U_{g}\right\rangle -\mathrm{RT}$$

where <*U*_hg_> is the interaction energy between the framework and adsorbate, <*U*_h_> is the framework energy, <*U*_g_> is the internal energy of the adsorbate, *R* is the gas constant, and *T* is the temperature of the system in Kelvin. Widom insertion method can be used to calculate <*U*_hg_> − <*U*_h_>. <*U*_h_> and <*U*_g_> were set to zero because the framework and adsorbate were rigid, and no internal degrees of freedom were considered. Therefore, the heat of adsorption was calculated by subtracting *RT* from the <*U*_hg_> value obtained from the Widom insertion simulation. All MC simulations were performed using the RASPA 2.0 package.^[^ ^24^ ^]^

The structural features such as largest cavity diameter (LCD), and void fraction were computed using the Zeo++ package.^[^ ^25^ ^]^ The accessible gravimetric surface area (AGSA) was computed using a probe molecule with a radius of 1.655 Å that is the *σ* value of nitrogen in the TraPPE model. And the accessible pore volumes were computed using a probe molecule with a radius of 0 Å.

Constant temperature and pressure (NPT) MD simulations were performed for 1 ns for 100 structures showing the highest methane working capacity from GCMC simulations under LNG–ANG conditions (*P* = 6 bar and *T* = 159 K for charging; *P* = 5 bar and *T* = 298 K for discharging). Following the MD simulation, the structures were further energy‐minimized while the simulation cell parameters were fixed (NVT ensemble). Nose–Hoover thermostat and barostat were used to maintain the temperature and pressure of the system with damping parameters of 0.1 and 1.0 ps, respectively. Velocity–verlet integrator was used to numerically integrate the Newton's equations of motion with 0.1 fs timesteps. Conjugated gradient and fire algorithms, as implemented in the LAMMPS simulation package (version Dec 12, 2018),^[^ ^26^ ^]^ were used for the energy minimization step and the energy minimization was carried out until the energy difference between subsequent cycles was less than 1.0 × 10^−6^ kcal mol^−1^. Bonded interactions between framework atoms were approximated based on the UFF4MOF force field, as reported by Coupry et al.,^[^ ^21b^ ^]^ and force field parameters were assigned using the LAMMPS interface python module developed by Boyd et al.^[^ ^27^ ^]^ The effect of Coulomb interaction was ignored during the simulation. All MD simulations were performed using LAMMPS.^[^ ^26^ ^]^ For methane uptakes of the top 50 materials after energy minimization by the NVT ensemble, GCMC simulations were performed for 10 000 initialization cycles followed by 10 000 production cycles.

### Machine Learning

The dataset (*N* = 2144) was first divided into training (*N* = 1715) and testing (*N* = 429) sets based on the diversity selection method from Simon and coworkers (Figure S11, Supporting Information).^[^ ^28^ ^]^ For the diversity selection method, five structural features (largest cavity diameter (LCD), pore limiting diameter (PLD), accessible gravimetric surface area (AGSA), density, and void fraction (VF)) and the heat of adsorption (Δ*H*_ads_) were used. The scikit‐learn package^[^ ^29^ ^]^ in Python was used to apply the gradient boosting regressor (GBR) method. The GBR method was used to train regression trees by minimizing arbitrary loss functions with optimized hyperparameters based on the scikit‐optimize module's sequential model‐based optimization method. The accuracy of the GBR method was validated using the mean absolute error of fivefold, 100‐times‐repeated cross‐validation. The optimized hyperparameters used for ML are listed in Table S7, Supporting Information. In the trained GBR model, the feature importance for predicting the adsorption uptake (or working capacity) in each condition could be obtained. The data and Python script used to reproduce the ML workflow are listed in the Supporting Information.

### Synthesis of DUT‐23(Co) and DUT‐23(Cu)

DUT‐23(Co) and DUT‐23(Cu) were synthesized according to previous literature.^[^ ^16^ ^]^ Co(NO_3_)_2_·6H_2_O and Cu(NO_3_)_2_·3H_2_O were purchased from Sigma‐Aldrich and used without further purification. H_3_(4,4″,4′‐benzene‐1,3,5‐triyl‐tribenzoate) (H_3_(btb)) and 4,4″‐bipyridine (bipy) were purchased from Alfa Aesar and used without further purification. To synthesize DUT‐23(Co), precursors of Co(NO_3_)_2_·6H_2_O (291 mg), H_3_(btb) (109 mg), and bipy (42 mg) were dissolved in 10 mL of DEF, and the mixture was transferred to a 100 mL autoclave reactor. The reaction for 48 h at 373 K produced large, dark‐purple crystals, which were then purified several times with dimethylformamide (DMF). To synthesize DUT‐23(Cu), precursors of Cu(NO_3_)_2_·3H_2_O (241 mg), H_3_(btb) (109 mg), and bipy (43 mg) were dissolved in a solution of DMF and ethanol (EtOH) (DMF/EtOH = 5 mL/5 mL) plus 2 drops of trifluoroacetic acid. The mixture was transferred to a 100‐mL autoclave reactor. The reaction for 20 h at 353 K provided green crystals, which were purified several times with DMF. For both samples, the solvent in which the synthesized samples were submerged was exchanged to absolute EtOH for the following supercritical CO_2_ dry on a Samdri‐PVT‐3D (tousimis, USA).

### Characterization and Gas Adsorption

PXRD was performed for the synthesized samples and the DUT‐23(Cu) after the cyclic adsorption experiment using a Rigaku Ultima IV (Rigaku Co., Japan). In situ XRD patterns of DUT‐23(Cu) were obtained at various temperatures between 298 and 159 K using a D/MAX‐2500 (Rigaku Co., Japan). For the measurement, the sample was loaded at 298 K, and the chamber was evacuated and cooled to 159 K at the rate of 5 K min^−1^. At chamber temperatures of 298, 258, 218, 178, and 159 K, the cooling was paused, and the temperature was maintained for at least 5 min to record the XRD of the sample. All XRD patterns were recorded at 3–45° with the scan speed of 2° min^−1^.

Nitrogen adsorption isotherms were obtained at 77 K using a 3Flex analyzer (Micromeritics Instruments, USA). Before each measurement, the sample dried on the supercritical CO_2_ dryer was delivered to the cell of the 3Flex analyzer and degassed under vacuum at room temperature for 1 h. Gravimetric BET surface areas were calculated using the measured nitrogen isotherms based on two consistency criteria.^[^ ^30^ ^]^

Methane adsorption isotherms at 298 K up to 65 bar and those at 159 K up to 6 bar were measured using a Belsorp‐HP (MicrotracBEL., Japan). Before each measurement, the sample dried on a supercritical CO_2_ dryer was delivered to the cell of the Belsorp‐HP and degassed under vacuum at room temperature for 1 h. To maintain the temperature of the sample cell, an ethanol cooling bath constituting liquid nitrogen as a cooling agent was used at 159 K, and a water bath system with a circulator was used at 298 K.

Kinetic data for methane adsorption on DUT‐23(Cu) were measured using Autosorb‐IQ (Anton Paar QuantaTec Inc., Austria) at 298 and 159 K. For the measurement, the sample was activated and the temperature of the sample cell was maintained in the same way as for high‐pressure methane adsorption.

Field emission scanning electron microscopy images of the DUT‐23(Cu) samples were recorded using a JEOL‐IT500HR (JEOL Ltd., Japan) before and after five cycles of the temperature pressure swing adsorption experiment between 159 and 298 K.

## Conflict of Interest

The authors declare no conflict of interest.

## Supporting information

Supporting Information

## Data Availability

The data that support the findings of this study are available from the corresponding author upon reasonable request.

## References

[1] a) Y. He , W. Zhou , G. Qian , B. Chen , Chem. Soc. Rev. 2014, 43, 5657; 24658531 10.1039/c4cs00032c

[2] a) K. R. Matranga , A. L. Myers , E. D. Glandt , Chem. Eng. Sci. 1992, 47, 1569;

[3] a) B. Li , H.‐M. Wen , W. Zhou , Jeff Q. Xu , B. Chen , Chem 2016, 1, 557;

[4] a) G. Chang , H. Wen , B. Li , W. Zhou , H. Wang , K. Alfooty , Z. Bao , B. Chen , Cryst. Growth Des. 2016, 16, 3395;

[5] T. Düren , L. Sarkisov , O. M. Yaghi , R. Q. Snurr , Langmuir 2004, 20, 2683. 15835137 10.1021/la0355500

[6] a) D. Alezi , Y. Belmabkhout , M. Suyetin , P. M. Bhatt , Ł. J. Weseliński , V. Solovyeva , K. Adil , I. Spanopoulos , P. N. Trikalitis , A.‐H. Emwas , M. Eddaoudi , J. Am. Chem. Soc. 2015, 137, 13308; 26364990 10.1021/jacs.5b07053 PMC4616230

[7] a) H. Wang , Q.‐L. Zhu , R. Zou , Q. Xu , Chem 2017, 2, 52;

[8] a) Y. G. Chung , E. Haldoupis , B. J. Bucior , M. Haranczyk , S. Lee , H. Zhang , K. D. Vogiatzis , M. Milisavljevic , S. Ling , J. S. Camp , B. Slater , J. I. Siepmann , D. S. Sholl , R. Q. Snurr , J. Chem. Eng. Data 2019, 64, 5985;

[9] a) S. Chong , G. Thiele , J. Kim , Nat. Commun. 2017, 8, 1539; 29146929 10.1038/s41467-017-01478-4 PMC5691151

[10] C. M. Simon , J. Kim , D. A. Gomez‐Gualdron , J. S. Camp , Y. G. Chung , R. L. Martin , R. Mercado , M. W. Deem , D. Gunter , M. Haranczyk , D. S. Sholl , R. Q. Snurr , B. Smit , Energy Environ. Sci. 2015, 8, 1190.

[11] a) S. Kayal , B. Sun , A. Chakraborty , Energy 2015, 91, 772;

[12] C. M. Simon , J. Kim , L.‐C. Lin , R. L. Martin , M. Haranczyk , B. Smit , Phys. Chem. Chem. Phys. 2014, 16, 5499. 24394864 10.1039/c3cp55039g

[13] G. Verma , S. Kumar , H. Vardhan , J. Ren , Z. Niu , T. Pham , L. Wojtas , S. Butikofer , J. C. Echeverria Garcia , Y.‐S. Chen , B. Space , S. Ma , Nano Res. 2021, 14, 512.

[14] D. A. Gómez‐Gualdrón , C. E. Wilmer , O. K. Farha , J. T. Hupp , R. Q. Snurr , J. Phys. Chem. C 2014, 118, 6941.

[15] Sturluson, A. , M. T. Huynh , A. R. Kaija , C. Laird , S. Yoon , F. Hou , Z. Feng , C. E. Wilmer , Y. J. Colón , Y. G. Chung , Mol. Simul. 2019, 45, 1082. 10.1080/08927022.2019.1648809 PMC6774364 31579352

[16] N. Klein , I. Senkovska , I. A. Baburin , R. Grünker , U. Stoeck , M. Schlichtenmayer , B. Streppel , U. Mueller , S. Leoni , M. Hirscher , S. Kaskel , Chemistry 2011, 17, 13007. 21956516 10.1002/chem.201101383

[17] A. Figini‐Albisetti , L. F. Velasco , J. B. Parra , C. O. Ania , Appl. Surf. Sci. 2010, 256, 5182.

[18] M. G. Martin , J. I. Siepmann , J. Phys. Chem. B 1998, 102, 2569

[19] a) H. S. Koh , M. K. Rana , A. G. Wong‐Foy , D. J. Siegel , J. Phys. Chem. C 2015, 119, 13451;

[20] a) M. K. Rana , H. S. Koh , H. Zuberi , D. J. Siegel , J. Phys. Chem. C 2014, 118, 2929;

[21] a) S. L. Mayo , B. D. Olafson , W. A. Goddard , J. Phys. Chem. 1990, 94, 8897;

[22] J. J. Potoff , J. I. Siepmann , AIChE J. 2001, 47, 1676.

[23] a) D. A. Gomez‐Gualdron , O. V. Gutov , V. Krungleviciute , B. Borah , J. E. Mondloch , J. T. Hupp , T. Yildirim , O. K. Farha , R. Q. Snurr , Chem. Mater. 2014, 26, 5632;

[24] D. Dubbeldam , S. Calero , D. E. Ellis , R. Q. Snurr , Mol. Simul. 2016, 42, 81.

[25] T. F. Willems , C. H. Rycroft , M. Kazi , J. C. Meza , M. Haranczyk , Microporous Mesoporous Mater. 2012, 149, 134.

[26] A. P. Thompson , H. M. Aktulga , R. Berger , D. S. Bolintineanu , W. M. Brown , P. S. Crozier , P. J. in 't Veld , A. Kohlmeyer , S. G. Moore , T. D. Nguyen , R. Shan , M. J. Stevens , J. Tranchida , C. Trott , S. J. Plimpton , Comp. Phys. Comm. 2022, 271, 10817.

[27] P. G. Boyd , S. M. Moosavi , M. Witman , B. Smit , J. Phys. Chem. Lett. 2017, 8, 357. 28008758 10.1021/acs.jpclett.6b02532 PMC5253710

[28] C. M. Simon , R. Mercado , S. K. Schnell , B. Smit , M. Haranczyk , Chem. Mater. 2015, 27, 4459.

[29] F. Pedregosa , G. Varoquaux , A. Gramfort , V. Michel , B. Thirion , O. Grisel , M. Blondel , P. Prettenhofer , R. Weiss , V. Dubourg , J. Vanderplas , A. Passos , D. Cournapeau , M. Brucher , M. Perrot , É. Duchesnay , J. Mach. Learn. Res. 2011, 85, 2825.

[30] a) K. S. Walton , R. Q. Snurr , J. Am. Chem. Soc. 2007, 129, 8552; 17580944 10.1021/ja071174k

