# Supplementary material for: Discovery of High‐Performing Metal–Organic Frameworks for On‐Board Methane Storage and Delivery via LNG–ANG Coupling: High‐Throughput Screening, Machine Learning, and Experimental Validation
Source: Adv Sci (Weinh). 2022 May 7;9(21):2201559. doi: 10.1002/advs.202201559 (PMC9313482; doi:10.1002/advs.202201559)
Supplement: Supplementary file 1 — Supporting Information [file ADVS-9-2201559-s001.pdf]

Supplemental Information: Discovery of high-performing metal–organic frameworks for methane storage via LNG-ANG coupling: high-throughput screening and experimental validation

Seo-Yul Kim,[1,3,§] Seungyun Han,[2,§] Seulchan Lee,[2] Jo Hong Kang,[1,4] Sunghyun Yun,[2] Wanje Park,[1] Min Woo Shin,[1] Jinyoung Kim,[1] Yongchul G. Chung,[2,*] and Youn-Sang Bae[1,**]

[1]Department of Chemical and Biomolecular Engineering, Yonsei University, 50 Yonsei-ro, Seodaemungu, Seoul 03722, South Korea
[2]School of Chemical Engineering, Pusan National University, Busan 46241, South Korea
[3]School of Chemical and Biomolecular Engineering, Georgia Institute of Technology, Atlanta, GA 30332, USA.
[4]Korea Institute of Industrial Technology, 55 Joga-ro, Jung-gu, Ulsan 44413, South Korea
§These authors contributed equally
*Correspondence: drygchung@gmail.com
**Correspondence: mowbae@yonsei.ac.kr

Table S1. Methane uptakes and working capacities of benchmarking MOFs.

Structure (Code name)	Methane Uptake (cm^3(STP) cm^{-3})			Working Capacity (cm^3(STP) cm^{-3})	
	5 bar/298 K	65 bar/298 K	6 bar/159 K	ANG	LNG-ANG
NU-125 (REWNEO)	44.3	227.5	391.9	183.2	347.6
Ni-MOF-74 (ORIVUI)	79.2	229.4	323.6	150.2	244.4
HKUST-1 (BODPAN)	62.7	226.3	365.9	163.6	303.2
PCN-14 (XITYOP)	91.5	215.5	317.6	123.9	226.1
NJU-Bai-43 (cg400449c si 001)	55.5	217.8	375.8	162.3	320.2
MOF-210 (CUSYAR)	13.5	143.6	347.9	130.1	334.4
NU-111 (MEHMET)	24.4	188.9	400.6	164.5	376.3
Al-soc-MOF-1 (MUZKAV)	44.3	205.7	397.2	161.4	352.9

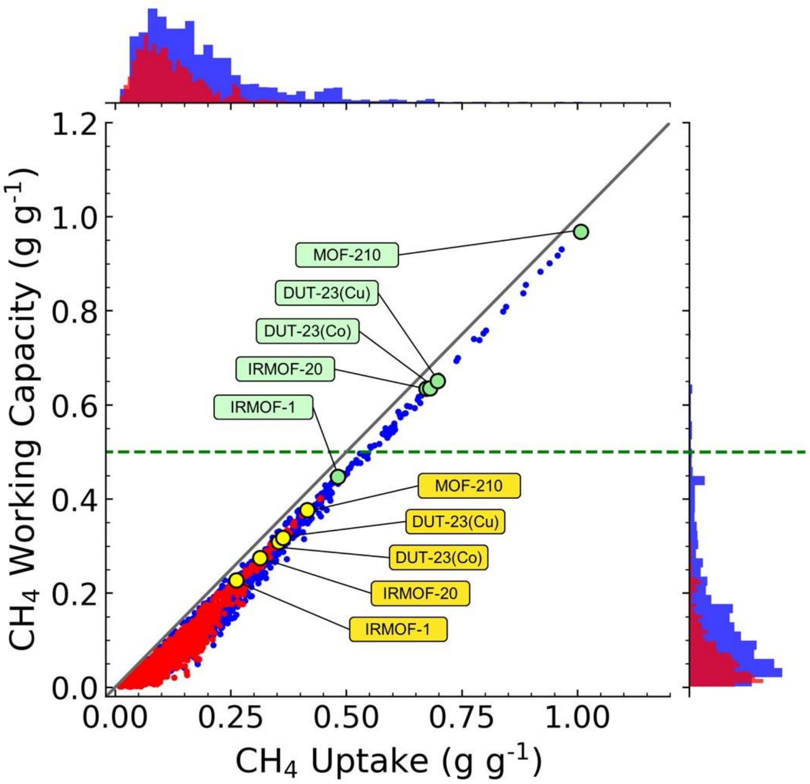

Figure S1. Gravimetric methane uptakes and working capacities from the high-throughput screening for the structures from the CoRE MOF 2019-ASR Database (N = 2,144) for LNG-ANG and ANG systems. Blue points and green circles represent simulation data from the LNG-ANG condition (159 K, 6 bar adsorption - 298 K, 5 bar desorption). Red points and yellow circles indicate simulation data from ANG condition (298 K, 65 bar adsorption - 298 K, 5 bar desorption). Green dashed lines highlight the ARPA-E targets (working capacity target: 0.5 g g^{-1}).

Table S2. Gravimetric methane uptakes and working capacities of DUT-23(Co) and DUT-23(Cu) calculated from GCMC simulation.

Structure (Code name)	Methane Uptake (g g^{-1})			Working Capacity (g g^{-1})		Rank*	Rank*
	5 bar/298 K	65 bar/298 K	6 bar/159 K	ANG	LNG-ANG	(gravimetric)	(volumetric)
DUT-23(Co) (ICAQIO)	0.046	0.36	0.70	0.32	0.65	18	46
DUT-23(Cu) (ICAQOU)	0.045	0.35	0.68	0.31	0.64	25	47

* The rank among total 2,144 structures.

Table S3. The ranges of structural properties of top 50 MOFs in adsorption condition of LNG-ANG and in adsorption condition of ANG. The average value standard deviation, first quartile, median value and third quartile are also given.

	LNG-ANG	ANG
AGSA (m^2 g^{-1})	3039 ~ 6787	2016 ~ 4800
	Avg. = 4090, Std. = 864	Avg. = 3814, Std. = 446
	Q1 = 3688, Mid = 3734, Q3 = 4711	Q1 = 3688, Mid = 3720, Q3 = 3768
Pore Volume (cm^3 g^{-1})	1.01 ~ 3.02	0.70 ~ 1.90
	Avg. = 1.67, Std. = 0.56	Avg. = 1.35, Std. = 0.16
	Q1 = 1.35, Mid = 1.37, Q3 = 2.04	Q1 = 1.35, Mid = 1.36, Q3 = 1.37
LCD (˚A)	10.9 ~ 25.2	7.5 ~ 17.3
	Avg. = 16.0, Std. = 3.3	Avg. = 13.8, Std. = 2.6
	Q1 = 13.1, Mid = 15.1, Q3 = 18.2	Q1 = 13.8, Mid = 15.0, Q3 = 15.1
Density (g cm^{-3})	0.287 ~ 0.729	0.437 ~ 1.074
	Avg. = 0.530, Std. = 0.136	Avg. = 0.603, Std. = 0.082
	Q1 = 0.409, Mid = 0.590, Q3 = 0.596	Q1 = 0.590, Mid = 0.595, Q3 = 0.599
Void Fraction	0.733 ~ 0.871	0.752 ~ 0.847
	Avg. = 0.811, Std. = 0.042	Avg. = 0.804, Std. = 0.015
	Q1 = 0.810, Mid = 0.810, Q3 = 0.838	Q1 = 0.802, Mid = 0.810, Q3 = 0.810
$\Delta H_{ads,298K}$ (kJ mol^{-1})	-14.1 ~ -7.8	-13.5 ~-9.2
	Avg. = -11.1, Std. = 1.6	Avg. = -10.7, Std. = 0.6
	Q1 = -11.5, Mid = -10.6, Q3 = -10.1	Q1 = -10.7, Mid = -10.6, Q3 = -10.5

*Avg. : Average , Std. : Standard deviation, Q1 : First quartile,
Mid : Median value, Q3 : Third quartile

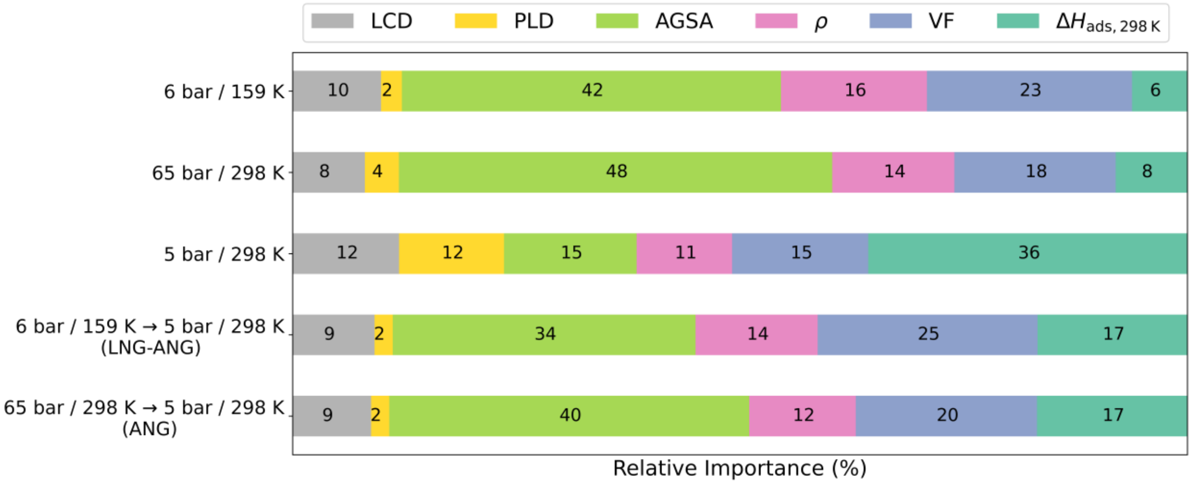

Figure S2. Relative feature importance for different operating conditions. Feature label definitions are: $\Delta H_{ads,298K}$ is the heat of adsorption (kJ mol^{-1}) at 298 K, VF is the void fraction, AGSA is the accessible gravimetric surface area (m^2 g^{-1}), ρ is the density of framework (g cm^{-3}), PLD is the pore limiting diameter (Å), and LCD is the largest cavity diameter (Å).

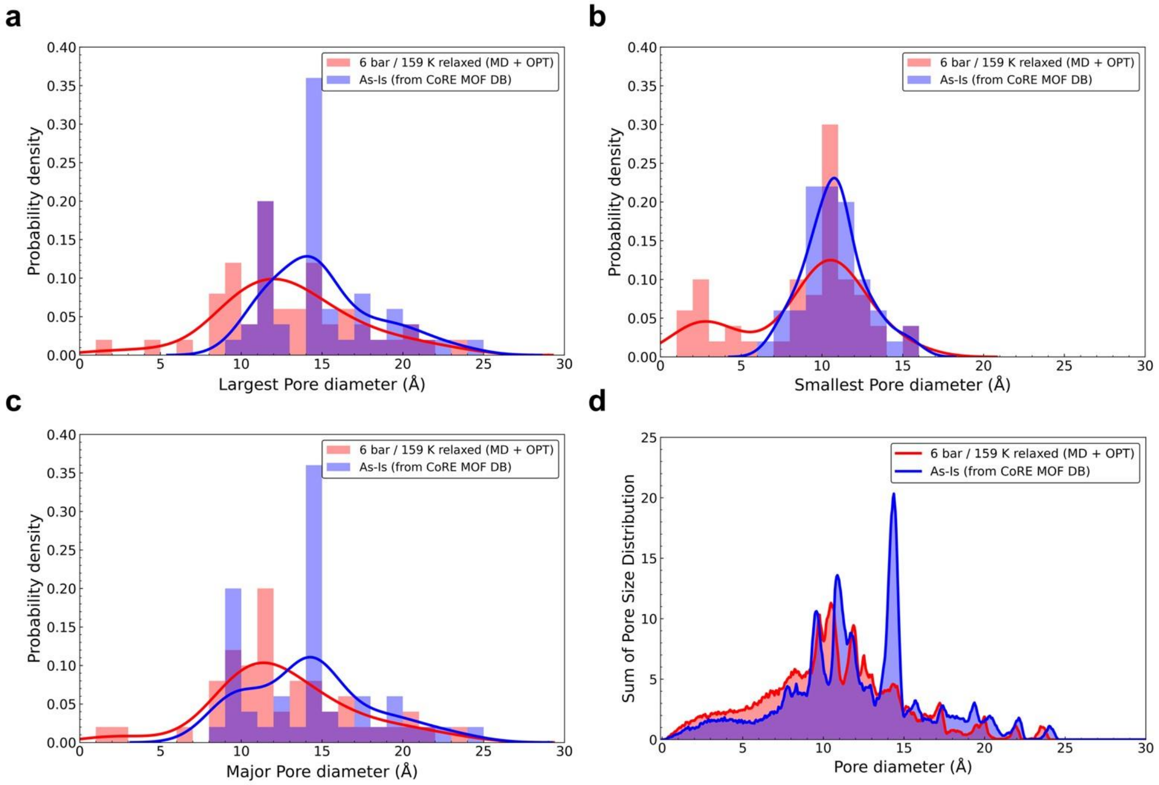

Figure S3. Histogram of (a) largest, (b) smallest, (c) major pore diameter among the peak of pore size distribution graph. Largest pore diameter is the diameter which marked the first peak from left side in each PSD. Smallest pore diameter is the diameter which marked the first peak from right side in each PSD. Major pore diameter is the diameter which marked the peak with the largest probability in each PSD. Plot (d) for sum of PSD to the diameter of each structure. 'MD + OPT' is result that final configuration from the NPT MD simulations were then energy minimized under the NVT ensemble.

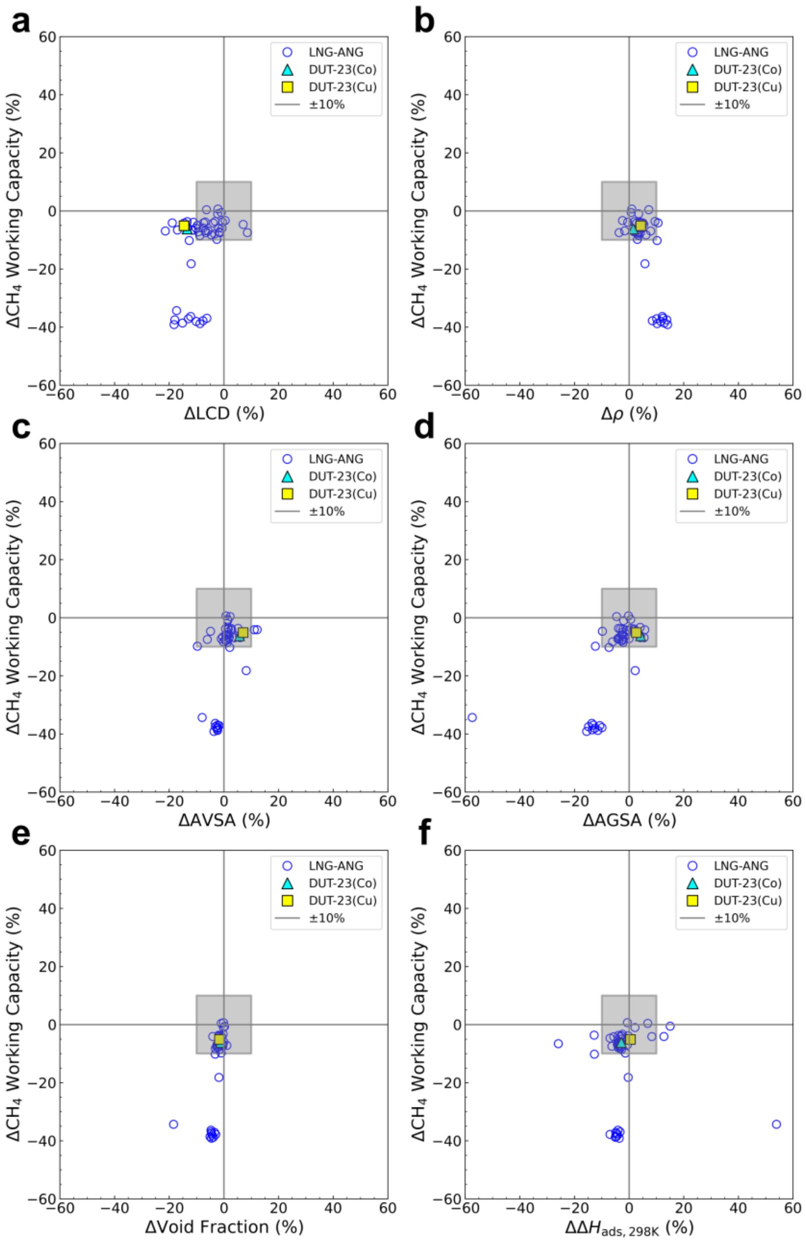

Figure S4. The effects of each property on the working capacity. The x-axis are the percent changes in (a) LCD, (b) density, (c) accessible volumetric surface area, (d) accessible gravimetric surface area, (e) void fraction and (f) heat of adsorption at 298 K of the top 50 materials from high-throughput screening after the MD simulation runs at 6 bar/159 K. The y-axis of is the percent changes in the methane working capacities under LNG-ANG condition.

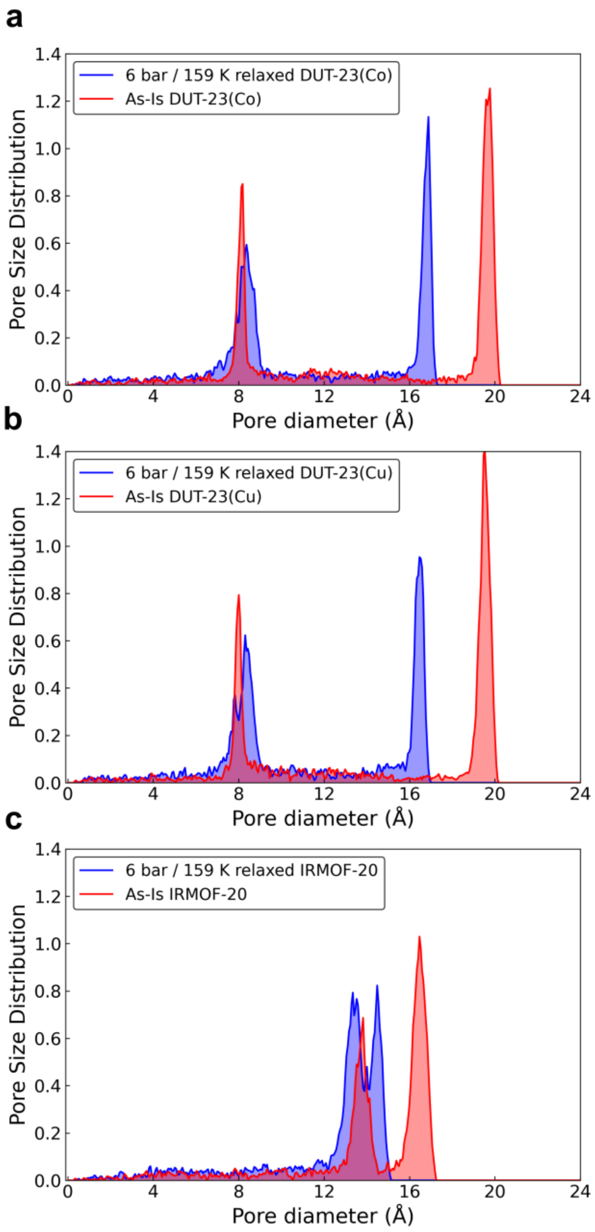

Figure S5. Pore size distribution of (a) DUT-23(Co), (b) DUT-23(Cu), (c) IRMOF-20.

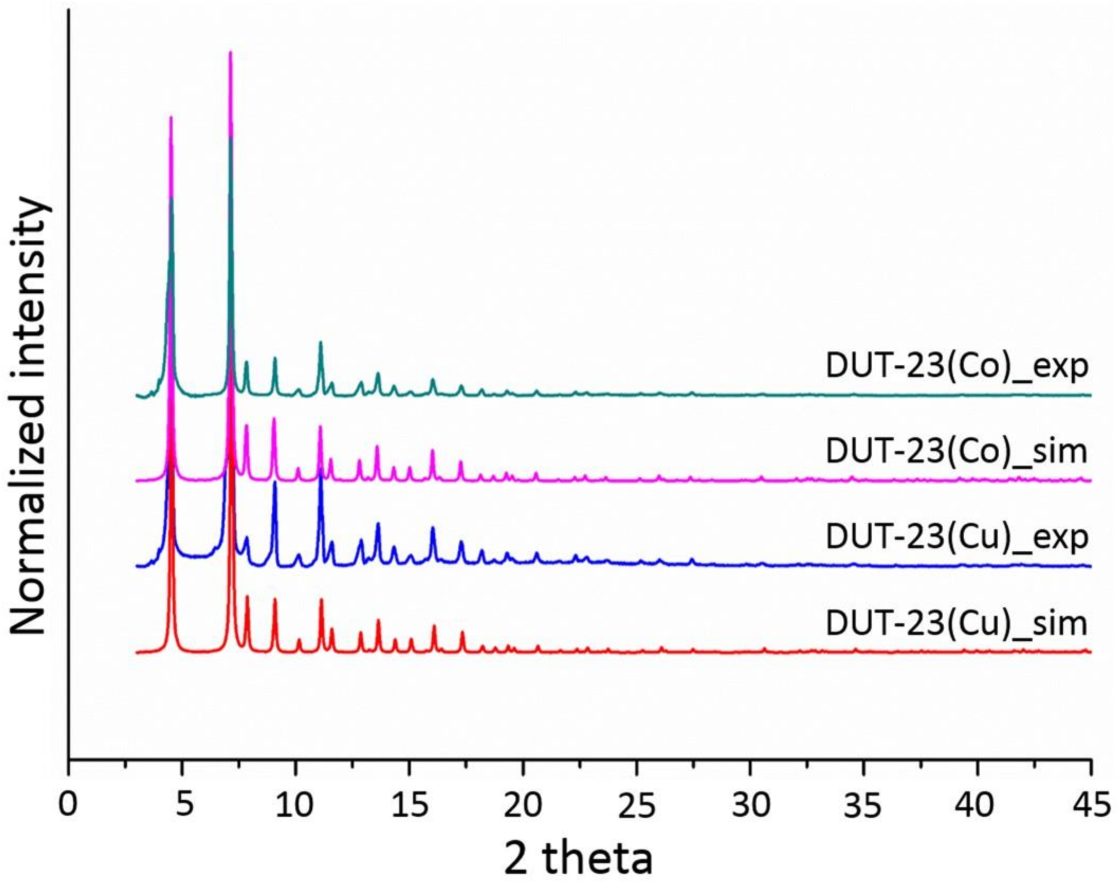

Figure S6. PXRD patterns for synthesized DUT-23(Co) and DUT-23(Cu), compared to those calculated from their structures in CoRE MOF database.

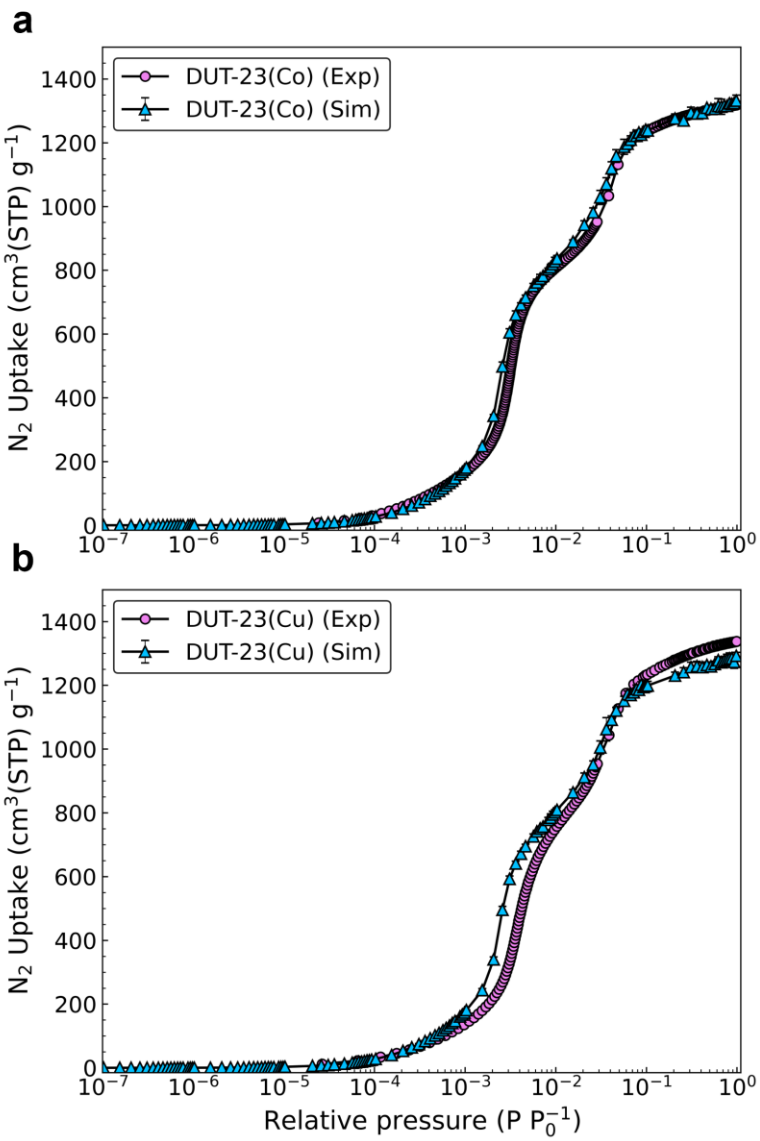

Figure S7. N_2 isotherms at 77 K for (a) DUT-23(Co) and (b) DUT-23(Cu) from experiments and from GCMC simulations. Details of the simulations are provided in the main text.

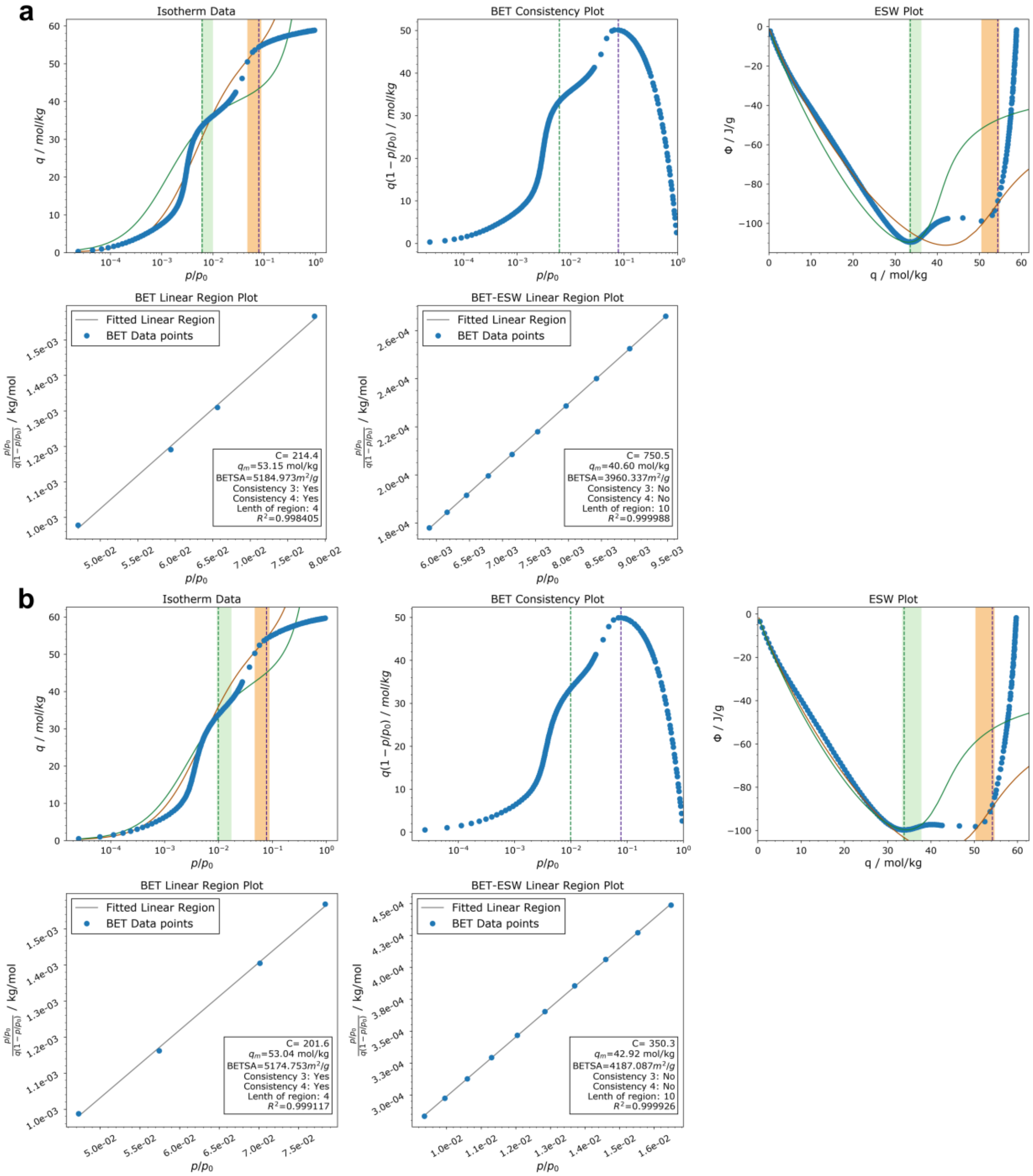

Figure S8. The BET analyses of (a) DUT-23(Co) and (b) DUT-23(Cu) from experimentally measured N_2 isotherms at 77 K using the SESAMI package.

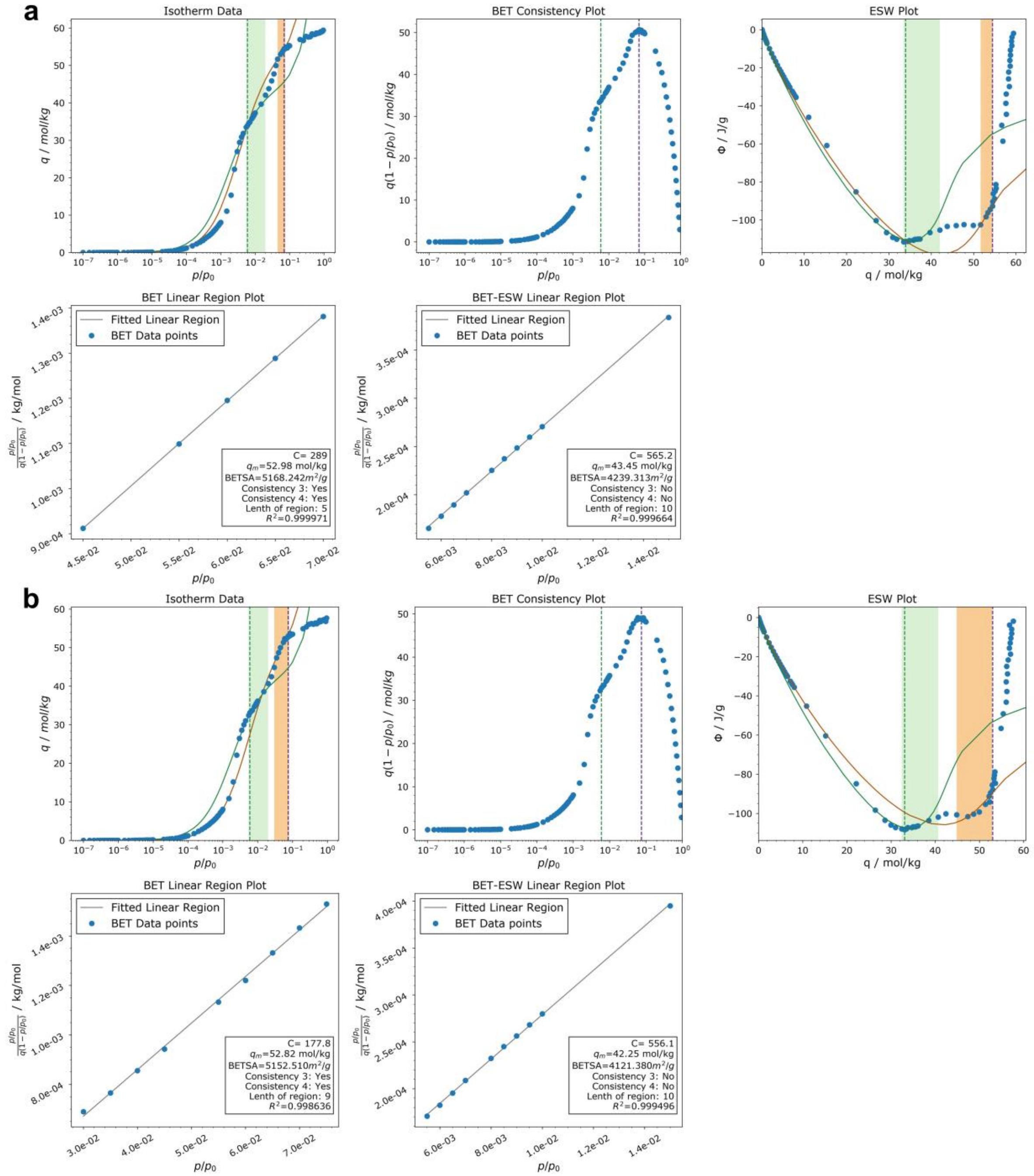

Figure S9. The BET analyses of (a) DUT-23(Co) and (b) DUT-23(Cu) from simulated N_2 isotherms at 77 K using the SESAMI package.

Table S4. Comparison of the BET and BET+ESW areas of DUT-23(M) (M = Cu, Co) computed from different N_2 isotherms.

MOF name	Case	BET (m^2g^{-1})	BET 3rd 4th	Geometric (m^2g^{-1})	BET+ESW (m^2g^{-1})	BET+ESW 3rd 4th
DUT-23(Co)	Experiment	5185	yes, yes		3960	no, no
	Simulation	5168		4746	4239	no, no
DUT-23(Cu)	Experiment	5175	yes, yes		4187	no, no
	Simulation	5152	yes, yes	4636	4121	no, no

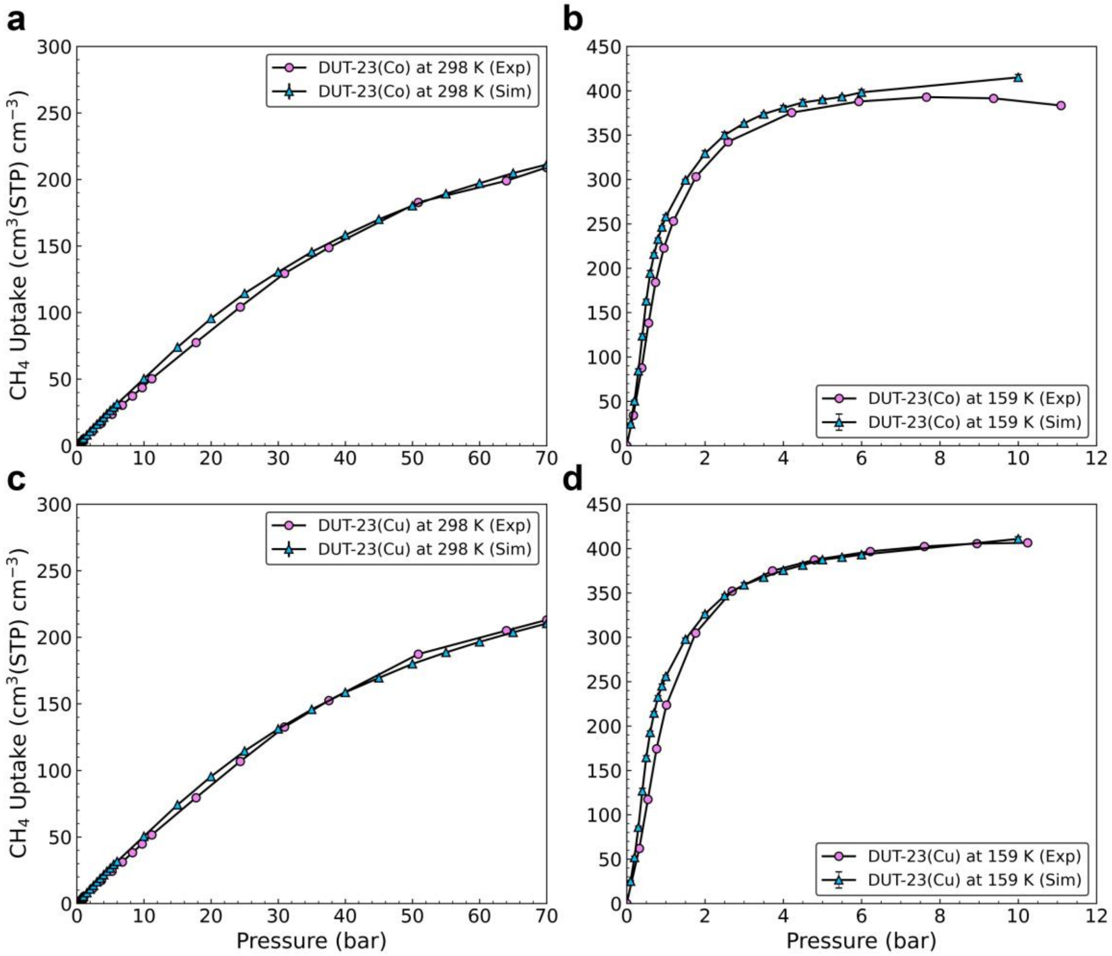

Figure S10. Comparison between simulated and experimentally measured methane isotherms for (a, b) DUT-23(Co) and (c, d) DUT-23(Cu).

Table S5. The Lennard-Jones parameters for the framework atoms.

Atom type	ϵ/κ_B (K)	σ (Å)	Atom type	ϵ/κ_B (K)	σ (Å)
Al	156	3.91	Mg	55.85	2.69
As	155.47	3.77	Mn	6.54	2.64
B	47.81	3.58	N	38.95	3.26
Ba	183.15	3.3	Na	15.09	2.66
Be	42.77	2.45	Nb	29.69	2.82
Bi	260.63	3.89	Ni	7.55	2.52
Br	126.29	3.73	Np	9.56	3.05
C	47.86	3.47	O	48.16	3.03
Ca	25.16	3.09	P	161.03	3.7
Cd	114.72	2.54	Pb	333.59	3.83
Ce	6.54	3.17	Rb	20.13	3.67
Cl	142.56	3.52	Re	33.21	2.63
Co	7.04	2.56	S	173.11	3.59
Cr	7.55	2.69	Sb	225.91	3.94
Cu	2.52	3.11	Sc	9.56	2.94
Cs	22.64	4.02	Si	156	3.8
Er	3.52	3.05	Sn	285.28	3.91
F	36.48	3.09	Sr	118.24	3.24
Fe	27.68	4.04	Ta	40.75	2.82
Ge	190.69	3.81	Th	13.08	3.03
H	7.65	2.85	Ti	8.55	2.83
Hg	193.71	2.41	V	8.05	2.8
Ho	3.52	3.04	W	33.71	2.73
I	170.57	4.01	Yb	114.72	2.99
Li	12.58	2.18	Zn	27.68	4.04
Lu	20.63	3.24	Zr	34.72	2.78

Table S6. Transferable Potential for Phase Equilibria (TraPPE) force field parameters for adsorbate molecules.

Atom type	ϵ/κ_B (K)	σ (Å)	q (e)
CH_4	148.0	3.73	0.0
N	36.0	3.31	-0.4048
N_2_COM	-	-	0.8096

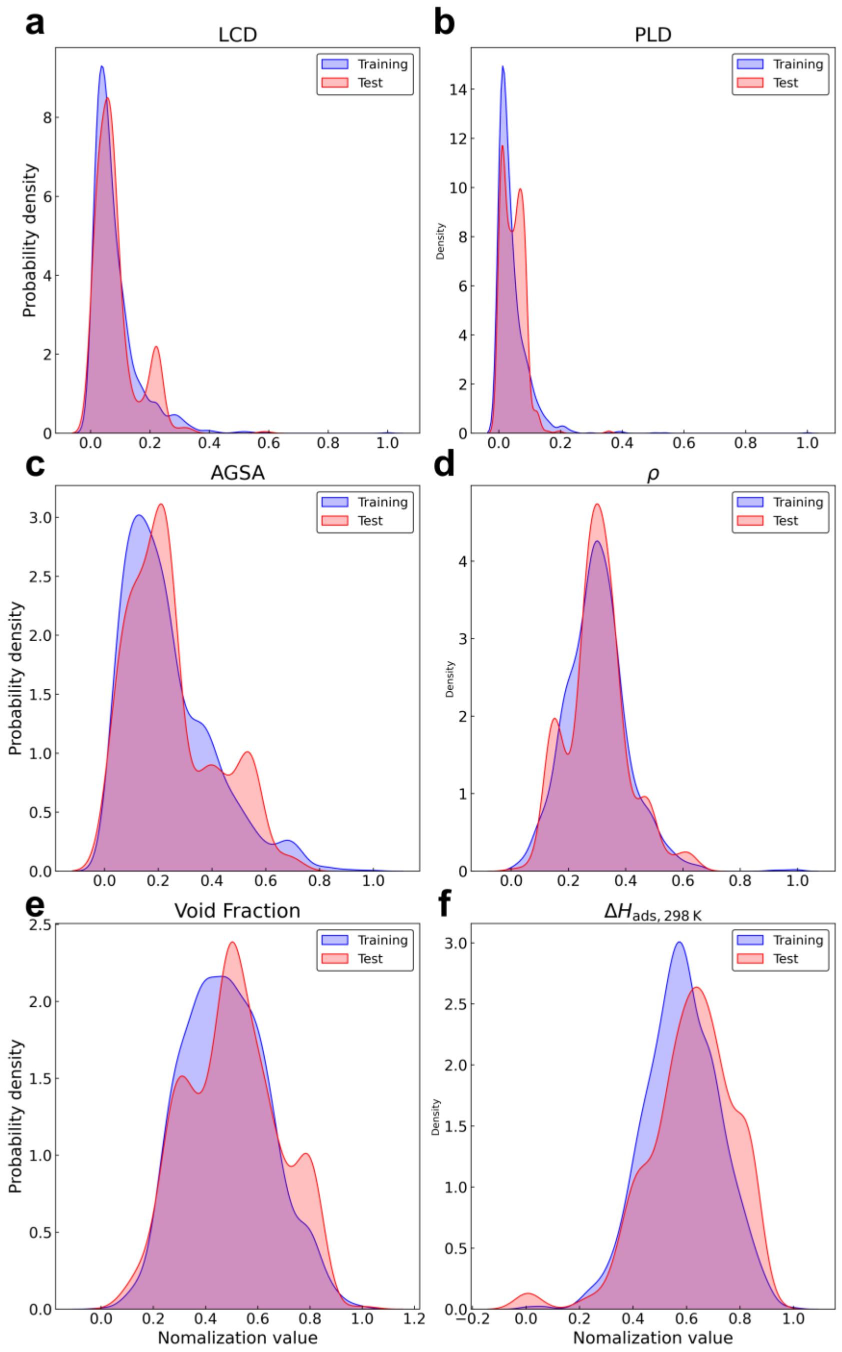

Figure S11. Histogram of structural properties used in machine learning. (a) the largest cavity diameter (Å), (b) pore limiting diameter (Å), (c) accessible gravimetric surface area (AGSA, m^2 g^{-1}), (d) density (g cm^{-3}), (e) void fraction, (f) the heat of adsorption (kJ mol^{-1}). The units are normalized with respect to the largest value from the dataset.

Table S7. Optimized hyperparameter of gradient-boosting regressor (GBR) model.

Parameter	Value				
	LNG-ANG	ANG	5 bar/298 K	6 bar/159 K	65 bar/298 K
estimators	975	1000	1000	925	1000
max depth	5	5	5	5	5
learning rate	0.020804	0.032787	0.023895	0.026347	0.03781
minimum samples split	2	2	2	2	2
minimum samples per leaf	1	1	1	1	1
max features	square root of total features				
R^2 (Training set)	0.992	0.996	0.980	0.992	0.994
R^2 (Test set)	0.977	0.987	0.945	0.974	0.969
RMSE (Training set)	8.538	2.943	4.065	7.558	3.466
RMSE (Test set)	16.103	5.951	7.060	15.245	8.159
MAE (Training set)	6.605	2.274	3.029	5.748	2.661
MAE (Test set)	10.397	4.476	4.173	10.019	5.959

*RMSE : Root Mean Square Error, MAE : Mean Absolute Error

Table S8. Optimized hyperparameter and accuracy of Random Forest model.

Parameter	Value				
	LNG-ANG	ANG	5 bar/298 K	6 bar/159 K	65 bar/298 K
estimators	864	1000	822	840	678
max depth	5	5	5	5	5
minimum samples split	2	2	2	2	2
minimum samples per leaf	1	1	1	1	2
max features	square root of total features				
R^2 (Training set)	0.942	0.942	0.653	0.916	0.824
R^2 (Test set)	0.944	0.951	0.736	0.918	0.834
RMSE (Training set)	22.441	11.331	16.889	25.117	19.514
RMSE (Test set)	24.795	11.595	15.476	26.944	18.888
MAE (Training set)	16.682	8.684	12.054	18.784	14.957
MAE (Test set)	17.198	8.900	9.497	19.280	14.948

*RMSE : Root Mean Square Error, MAE : Mean Absolute Error

Table S9. Optimized hyperparameter and accuracy of Decision Tree model.

Parameter	Value				
	LNG-ANG	ANG	5 bar/298 K	6 bar/159 K	65 bar/298 K
max depth	5	5	5	5	5
minimum samples split	2	11	2	2	2
minimum samples per leaf	11	7	1	1	1
max features	square root of total features				
R^2 (Training set)	0.906	0.918	0.577	0.891	0.784
R^2 (Test set)	0.934	0.943	0.672	0.882	0.800
RMSE (Training set)	28.578	13.437	18.653	28.596	21.618
RMSE (Test set)	26.955	12.428	17.257	32.328	20.761
MAE (Training set)	21.005	10.311	13.398	21.896	16.576
MAE (Test set)	19.385	9.907	10.717	23.552	16.385

*RMSE : Root Mean Square Error, MAE : Mean Absolute Error

Table S10. Optimized hyperparameter and accuracy of Support Vector Machine model.

Parameter	Value				
	LNG-ANG	ANG	5 bar/298 K	6 bar/159 K	65 bar/298 K
C	10^5	10^3	10^4	10^4	10^4
Gamma	10^{-6}	10^{-5}	10^{-6}	10^{-6}	10^{-5}
kernel	RBF				
max iteration	100000				
R^2 (Training set)	0.870	0.900	0.620	0.848	0.778
R^2 (Test set)	0.903	0.937	0.641	0.864	0.787
RMSE (Training set)	33.655	14.883	17.675	33.775	21.938
RMSE (Test set)	32.776	13.081	18.052	34.640	21.409
MAE (Training set)	21.887	10.851	12.333	23.666	15.913
MAE (Test set)	22.448	10.047	12.098	24.314	15.283

*RMSE : Root Mean Square Error, MAE : Mean Absolute Error

Table S11. Multiple linear regression equation for working capacities and methane uptakes in LNG-ANG and ANG conditions derived from Lasso model.

Parameter	Value				
	LNG-ANG	ANG	5 bar/298 K	6 bar/159 K	65 bar/298 K
alpha	0.01	0.01	0.01	0.01	0.01
weight 1 (LCD term coeff.)	2.98E+00	5.44E-01	-3.55E+00	-5.90E-01	-3.03E+00
weight 2 (PLD term coeff.)	-2.56E+00	-1.17E+00	-2.56E-01	-2.82E+00	-1.44E+00
weight 3 (AGSA term coeff.)	1.34E-02	6.00E-03	-1.00E-03	1.20E-02	4.64E-03
weight 4 (density term coeff.)	1.42E+01	3.29E+00	3.30E+00	1.80E+01	7.10E+00
weight 5 (VF term coeff.)	5.54E+02	2.82E+02	1.97E+02	7.58E+02	4.87E+02
weight 6 (Heat term coeff.)	4.05E+00	2.41E+00	-5.14E+00	-1.13E+00	-2.77E+00
intercept	-145.165	-51.939	-115.243	-265.144	-171.918
R^2 (Training set)	0.884	0.866	0.443	0.855	0.675
R^2 (Test set)	0.890	0.909	0.421	0.819	0.652
RMSE (Training set)	31.810	17.195	21.398	32.975	26.524
RMSE (Test set)	34.896	15.706	22.935	40.049	27.343
MAE (Training set)	22.276	12.736	16.270	23.615	20.357
MAE (Test set)	23.572	11.787	17.765	28.208	20.271

*RMSE : Root Mean Square Error, MAE : Mean Absolute Error
